# Evaluation of Treatment Thresholds for Unconjugated Hyperbilirubinemia in Preterm Infants: Effects on Serum Bilirubin and on Hearing Loss?

**DOI:** 10.1371/journal.pone.0062858

**Published:** 2013-05-07

**Authors:** Christian V. Hulzebos, Paula van Dommelen, Paul H. Verkerk, Peter H. Dijk, Henrica L. M. Van Straaten

**Affiliations:** 1 Division of Neonatology, Department of Pediatrics, Beatrix Children’s Hospital, UMC Groningen, Groningen, The Netherlands; 2 Department of Behavioural and Societal Sciences, TNO, Leiden, The Netherlands; 3 Division of Neonatology, Department of Pediatrics, Isala Clinics Zwolle, Zwolle, The Netherlands; Hôpital Robert Debré, France

## Abstract

**Background:**

Severe unconjugated hyperbilirubinemia may cause deafness. In the Netherlands, 25% lower total serum bilirubin (TSB) treatment thresholds were recently implemented for preterm infants.

**Objective:**

To determine the rate of hearing loss in jaundiced preterms treated at high or at low TSB thresholds.

**Design/Methods:**

In this retrospective study conducted at two neonatal intensive care units in the Netherlands, we included preterms (gestational age <32 weeks) treated for unconjugated hyperbilirubinemia at high or low TSB thresholds. Infants with major congenital malformations, syndromes, chromosomal abnormalities or toxoplasmosis, rubella, cytomegalovirus, herpes, syphilis, and human immunodeficiency infections were excluded. We analyzed clinical characteristics and TSB levels during the first ten postnatal days. After two failed automated Auditory Brainstem Response (ABR) tests we used the results of the diagnostic ABR examination to define normal, unilateral, and bilateral hearing loss (>35 dB).

**Results:**

There were 479 patients in the high and 144 in the low threshold group. Both groups had similar gestational ages (29.5 weeks) and birth weights (1300 g). Mean and mean peak TSB levels were significantly lower after the implementation of the novel thresholds: 152±43 µmol/L and 212±52 µmol/L versus 131±37 µmol/L and 188±46 µmol/L for the high versus low thresholds, respectively (P<0.001). The incidence of hearing loss was 2.7% (13/479) in the high and 0.7% (1/144) in the low TSB threshold group (NNT = 50, 95% CI, 25–3302).

**Conclusions:**

Implementation of lower treatment thresholds resulted in reduced mean and peak TSB levels. The incidence of hearing impairment in preterms with a gestational age <32 weeks treated at low TSB thresholds was substantially lower compared to preterms treated at high TSB thresholds. Further research with larger sample sizes and power is needed to determine if this effect is statistically significant.

## Introduction

In 2007, lower treatment thresholds were introduced for the management of unconjugated hyperbilirubinemia in Dutch preterm infants of less than 32 weeks of gestation. [1] Universal aim of these guidelines for jaundiced preterm infants is to reduce the incidence of hyperbilirubinemia and bilirubin neurotoxicity. However, legitimate evidence on harmful total serum bilirubin (TSB) levels and safe TSB thresholds in preterm infants is lacking.[2]–[6] Auditory dysfunction has been observed in preterm neonates at TSB levels below exchange transfusion thresholds. It is known that the neural auditory pathway is one of the most sensitive areas of the central nervous system for bilirubin neurotoxicity. [7] Free unconjugated bilirubin (UCB_free_), the fraction of UCB not bound to plasma proteins (mainly albumin), can translocate across the blood-brain barrier and cause neurotoxicity. In line with this is the increased likelihood of an abnormal hearing screening test, i.e. an automated auditory brainstem response (AABR), with increasing UCB_free_ levels in preterm infants of 34 or more weeks of gestation. [8], [9] In these late preterm infants, no relation was demonstrated between an abnormal AABR and the TSB level. In younger preterm infants, the exact relation between TSB levels and hearing loss, including AABR, complete audiologic examination, and a diagnostic ABR is largely unknown. These preterm infants are especially prone for impeding bilirubin neurotoxicity and subtle ABR changes have been demonstrated at rather low TSB levels of 170 µmol/L. [10], [11] Introduction of novel consensus – based management guidelines of preterm infants with unconjugated hyperbilirubinemia in 2007 enabled us to compare the incidence of hearing impairment in jaundiced preterm infants treated at high TSB thresholds or at low TSB thresholds.

In addition, the effect of a variety of demographic and clinical characteristics on hearing impairment was analyzed.

## Methods

### Ethics Statement

According to the Research involving Human Subjects Act (WMO), because this study only includes retrospective research with data, it is not subject to an ethics review and does not need to include informed consent from the subjects, as the data was used anonymously. This study fulfils all the requirements for test subject anonymity and is in accordance with regulations of the review boards of the participating hospitals.

### Patients

Preterm infants of less than 32 weeks of gestation were included when admitted to one of the two participating NICU centers in two time periods: between January 2005 and December 2006, i.e. before introduction of the novel thresholds, and between March 2007 and March 2008. Exclusion criteria consisted of major congenital malformations, clinical syndromes, chromosomal abnormalities or TORCH (toxoplasmosis, rubella, cytomegalovirus, herpes, syphilis, and human immunodeficiency viral). These infections were excluded as these conditions are likely to affect hearing status. In addition, infants who died before the automated auditory brainstem response (AABR) was carried out were excluded.

### Treatment

Eligible infants admitted in 2005 and 2006 to one of the participating NICU centers were included and used as a control group: the high threshold group. In this group, treatment of unconjugated hyperbilirubinemia was applied according to identical guidelines for four (birth weight) categories: <1000 grams, 1000–1500 grams, 1500–2500 grams, and term newborns. In April 2007, a new “consensus based” guideline for preterm infants was implemented in the Netherlands. Treatment thresholds were ∼25% lower in the novel guideline. [1], [12] Eligible infants between April 2007 and March 2008 were included in this low threshold group. Five birth weight categories were used: <1000 grams, 1000–1250 grams, 1250–1500 grams, 1500–2000 grams and >2000 grams. In both treatment groups, preterm infants were classified as standard or high risk depending on the presence of commonly applied risk factors: asphyxia (defined as an Apgar Score <3 after 5 minutes), hypoxemia (paO2<5.3 kPa for more than 2 hours in the last 24 hours), acidosis (pH<7.15 for more than 1 hour in the last 24 hours), hemolysis (as demonstrated by a positive Coombs’ reaction), extreme low birth weight (birth weight <1000 grams), sepsis with circulatory insufficiency, meningitis, and intracranial haemorrhage (ICH) grade 2 or more according to Papile. In both centers, irradiance of phototherapy light sources was measured to comply with the international recommended intensity of phototherapy (center 1∶10.5±2.5 µW/cm^2^/nm; center 2∶7.4±3.3 µW/cm^2^/nm; p = 0.11). [13].

### Bilirubin Testing

Total serum bilirubin was tested in the first 10 postnatal days or when clinically indicated. Bilirubin measurements were performed using routine laboratory techniques. In each infant, the mean and mean peak of TSB measurements were calculated and used for analysis.

### Hearing Testing

All Dutch NICUs participate in a two-stage AABR neonatal hearing programme. The instruments’ algorithm of the AABR assumes an infant as passing the test when the acquired data obtained at 35 dB fits with 99.96% likelihood a template composed of auditory brainstem responses from normal hearing newborns. [14] Infants who failed the two AABR stages were referred to an audiological centre for further diagnostics, including conventional ABR, considered as the gold standard for detection of hearing level. The result of ABR was considered impaired when the infant’s ABR hearing levels exceeded 35 dB in at least one ear. Those infants who passed the screening were assumed to have normal hearing thresholds at that time.

### Statistics

Descriptive analyses were performed to determine the incidence of hearing loss and background characteristics in the high and low threshold group. Sensitivity and specificity of mean and mean peak TSB levels were evaluated using receiver-operating characteristics (ROC) curves.

## Results

**Figure 1:**
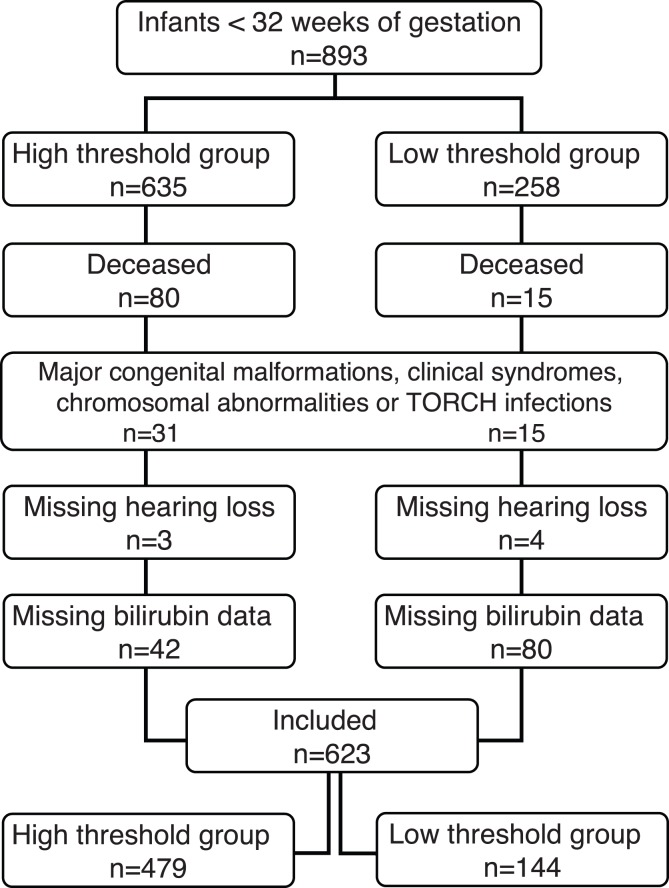
Flow chart with included and excluded infants of both NICU centers.

Figure 1 shows the number of included infants. The eligible cohort existed of 893 infants. A substantial number 270 (30%) was excluded, most often due to the absence of any bilirubin measurements (14%), death (11%), or predefined exclusion criteria (5%). We studied 623 (70%) of preterm infants who were admitted during the study period, 144 were treated at low thresholds and 479 at high thresholds, respectively. The infants of the low and high thresholds group were similar with respect to gestational age and birth weight (Table 1). Infectious complications, i.e., sepsis and meningitis, were more often diagnosed in preterm infants treated according to the novel bilirubin thresholds.

**Table 1:** Clinical characteristics of jaundiced preterm infants <32 weeks of gestation stratified by high and low TSB treatment thresholds.

	High TSBthresholdsn = 479	Low TSB thresholdsn = 144
Gestational age, wks	29.5±1.7	29.5±1.6
Birth weight, g	1300±350	1310±310
Apgar score at 1′	7 [0–10]	6 [1]–[10]
Mechanical ventilation, n (%)	273 (57)	93 (65)
Sepsis, n (%)	163 (34)	82 (57)*
Meningitis, n (%)	7(2)	9 (6)*
Intracerebral bleeding, n (%)	15 (3)	4 (3)
Intensive care, days	16 [2–121]	17 [3–100]
Intensive care ≥ 12 days, n (%)	310 (65)	103 (72)
TSB mean, µmol/L	152±43	131±37*
TSB max, µmol/L	212±52	188±46*
Postnatal age of TSB max, day#	5.2±1.7	4.7±1.7*
No hearing loss, n/N (%)	466/479 (97.3)	143/144 (99.3)
Hearing loss, n/N (%)	13/479 (2.7)	1/144 (0.7)

Data represent mean (± SD) or median [ranges];

# data are based on 453 and 137 infants, resp.;

* P<0.005.

**Figure 2:**
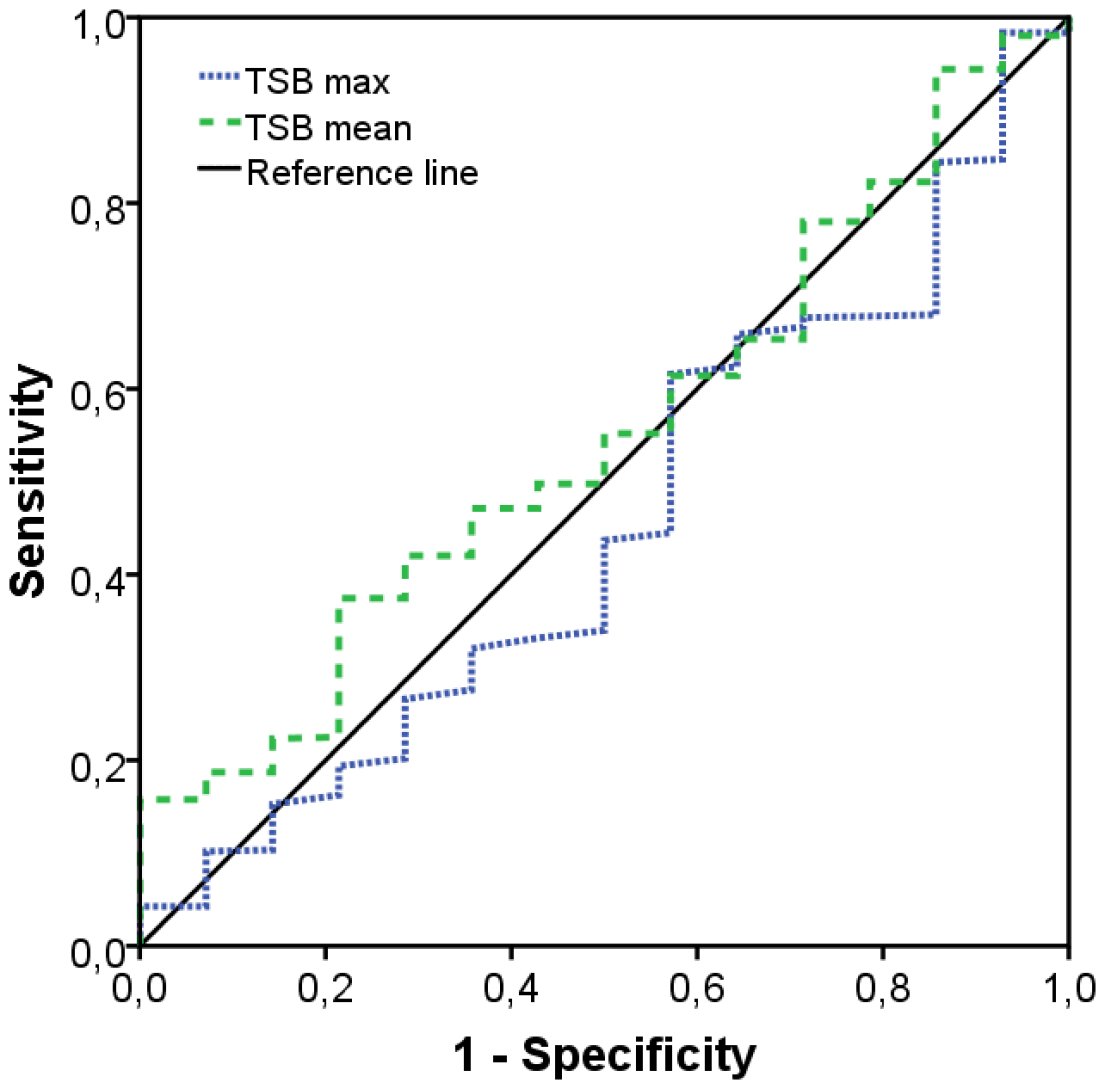
ROC curves of TSB levels as predictor of hearing loss.

No difference was observed in the proportion of infants who were hospitalized ≥ 12 days at intensive care, which is seen as an independent risk indicator for hearing loss. [15] As shown in Table 1, preterm infants of the two threshold groups showed significant differences in mean and mean peak TSB levels (P<0.001). Mean and mean peak TSB levels were ∼20 µmol/L and 25 µmol/L lower in preterm infants of the low threshold group. The incidence of hearing loss was substantially higher in preterm infants treated at high TSB thresholds (2.7%) compared to infants treated at low TSB thresholds (0.7%), resulting in a Number Needed to Treat of 50 (95% CI: 25–3302). The difference was not statistically significant (P = 0.21), probably because of insufficient power. Consanguinity was not present in infants with hearing loss. A positive familial history of hearing loss was found in one infant (a brother of the mother) with hearing loss in the high threshold group (NS). Peak TSB levels occurred significantly earlier (∼0.5 days) in preterm infants of the low threshold group. To assess whether a specific TSB level existed to predict hearing loss, ROC curves were constructed for mean TSB and mean peak TSB levels. Figure 2 shows that neither mean TSB level nor mean peak TSB level is a robust predictor of hearing loss: both areas under the curve (AUC) were approximately 0.5. In agreement with this we found similar mean and mean peak TSB levels in infants treated without (n = 609) and with (n = 14) hearing loss (148±43 µmol/L vs. 139±40 µmol/L and 206±52 µmol/L vs. 210±58 µmol/L; no hearing loss vs. hearing loss, resp.).

## Discussion

This retrospective cohort study demonstrates that introduction of lower treatment thresholds in preterm infants results in significantly lower mean and mean peak TSB levels. Treatment according to novel treatment thresholds did not result in a significant reduction of hearing impairment, albeit a ∼75% reduction in the incidence of hearing loss. Finally, and in analogy to previous data in term infants, it appeared that TSB level is also a poor predictor of hearing loss in preterm infants.

In 2007, the Dutch Society of Pediatrics approved and recommended a novel, “consensus-based” guideline on unconjugated hyperbilirubinemia in preterm infants. [1] Similar to other two existing guidelines of the United Kingdom and Norway, and the recently published “approach” of an expert committee in the USA, management of jaundiced preterm infants is now more uniform. [3], [16], [17] However, legitimate evidence on these applied treatment thresholds is lacking: to the best of our knowledge, long-term data on auditory or neurological outcome treated according to these guidelines are not at hand. As the auditory pathway is highest susceptible to bilirubin neurotoxicity, we chose next to bilirubin levels, the incidence of hearing impairment as a tool to evaluate the effectiveness of the novel Dutch guideline.

Infants treated according to the ∼25% lower thresholds had lower TSB levels: we found a reduction of ∼15% in mean and mean peak TSB levels, supporting an effective implementation of the new guideline.

Yet, auditory impairment in preterm infants may also occur at TSB levels below applied treatment thresholds, and - despite the rather low TSB levels- similar TSB levels have contributed to profound neurological impairment including sensoneurinal hearing loss in extreme or very low birth weight infants.[4], [18]–[20].

The incidence of hearing loss in the control group treated at higher TSB thresholds was in agreement with previous data: in a representative NICU population (10830 neonates with a mean (SD) gestational age 28.5 (1.6) weeks and birth weight of 1039 (256) g) the prevalence of hearing loss was 3.2% (95%-CI 2.6–4.1). [21] The incidence of hearing loss in preterm infants treated according to lower treatment thresholds was lower, whereas peak bilirubin levels occurred ∼0.5 days earlier and the clinical characteristics of these preterm neonates were significantly worse: infectious diseases occurred more often in this particular group. It is well known that sepsis and meningitis can result in severe neurodevelopmental abnormalities, including hearing loss. It is therefore tempting to speculate on the “protective” role of these novel, and lower treatment thresholds for jaundiced preterm infants. In agreement with this is data on high TSB levels which are known to significantly increase the risk of hearing loss, [22] although considerable overlap in TSB levels between very low birth weight infants with and without sensoneurinal hearing impairment has been reported. [18] The broad range in TSB levels in infants with hearing loss in our study and the occurrence of auditory impairment in preterm infants whose TSB levels never exceeded TSB thresholds, illustrate that TSB only is not a sensitive parameter to predict hearing loss, but that - for that matter - free bilirubin (Bf) may act as an important pathophysiological co-determinant of audiological dysfunction. In a retrospective case-control study in preterm and term infants (gestational ages 24–42 weeks) the role of Bf on audiologic dysfunction was delineated. [9] An abnormal AABR was associated with elevated levels of Bf and – even stronger with an elevated Bf/TSB ratio, which reflects the possibility of bilirubin entering the brain (i.e., Bf), and also the total bilirubin load of the infant (i.e., TSB). Data from the Neonatal Research Network confirmed the deleterious neurotoxic effects of Bf extremely low birth weight infants. [23] Levels of Bf would thus have been very informative, but were unavailable in our study. We acknowledge few other limitations: First, data on the postnatal course of TSB levels and applied treatment (phototherapy and exchange transfusion) are missing. Thus, the existence of prolonged bilirubin toxicity can only be speculated upon, whereas prolonged elevated TSB levels may pose an additional risk of ototoxicity in VLBW preterm infants. [24] Secondly, the retrospective character of this relative small study and concomitant selection bias does not provide sufficient power to adopt or adapt current treatment thresholds. Approximately 750 infants per group are necessary to detect a statistical significance difference between the reported prevalences of 2.7% and 0.7% (with an alpha of 0.05, and power of 80%). We are therefore cautious to claim any evidence on the applied treatment thresholds for preterm infants.

Despite these limitations, this is the first study to report on evaluation of a consensus-based national guideline in preterm infants with unconjugated hyperbilirubinemia regarding TSB levels and - unlike other studies - a formal diagnostic ABR (and not a screening ABR) as outcome parameters.

We conclude that successful implementation of lower TSB thresholds results in a reduction of mean TSB and mean peak TSB levels, and that larger studies - which preferably also provide data on Bf levels - are needed to establish a statistically significant effect of hyperbilirubinemia on hearing loss in very preterm infants.
